# Occupational factors and low back pain: a cross-sectional study of Bangladeshi female nurses

**DOI:** 10.1186/s13104-017-2492-1

**Published:** 2017-04-28

**Authors:** Shubrandu S. Sanjoy, Gias U. Ahsan, Hayatun Nabi, Ziaul F. Joy, Ahmed Hossain

**Affiliations:** 1 Department of Public Health, North South University, Bashundhara, Dhaka, 1229 Bangladesh; 2 Department of Genetic Engineering & Biotechnology, Shahjalal University of Science and Technology, Sylhet, Bangladesh

**Keywords:** Low back pain, Hospital occupational factors, Nurses, Bangladesh

## Abstract

**Background:**

The suffering from low back pain (LBP) is very common among nurses. The high prevalence rates of LBP are observed in many countries. Many back injuries are due to individual and work-related factors. Our aim is to investigate whether there is an association of occupational factors with LBP among the female nurses who are currently working in tertiary hospitals of Bangladesh.

**Methods:**

We conducted a cross-sectional study with 229 female nurses from two selected tertiary hospitals in Bangladesh. Data was collected through face-to-face interview using a standard structured questionnaire on four different measures of LBP along with questions on socio-demographic, occupational factors, physical and psychological factors.

**Results:**

Prevalence rates of LBP that lasted for at least 1 day, chronic LBP, intense pain and sought medical care because of LBP during the last 12 months were 72.9, 31.8, 24.4 and 36.2%, respectively. The multiple logistic regression analyses indicates that insufficient supporting staffs, overtime working hours and manual lifting in a working environment are associated with LBP. Besides, age and parity are found positively associated with chronic LBP.

**Conclusion:**

The prevalence of LBP among nurses in Bangladesh is high and should be actively addressed. Certain occupational factors play a key role in developing LBP among nurses. Nurses to patients ratio should be taken into consideration to reduce the occurrence of LBP among nurses employed in hospitals.

## Background

Low back pain (LBP) is defined as pain or discomfort, which also known as lumbago, occurs below the costal margin and above the gluteal fold [1, 2]. LBP is one of the leading public health problems with over 80% of the world population reporting LBP at some point in their life [3]. The prevalence of LBP is high especially in low and middle-income countries [4–6].

LBP is a common cause of morbidity in hospital health care workers. Nursing personnel, among the occupational groups within the health service, is more vulnerable to LBP [7]. A study in the USA indicates that nursing is one of the riskiest occupations for back pains, and it has the highest incidence of all types of nonfatal work-related injuries [8]. The risk factors of LBP among nurses are usually multifactorial, probably because the job needs a mixture of physically (such as manual handling of patients or medical equipment) and mentally demanding tasks (such as dealing with a crisis). Westgaard also discussed the effect of physical and mental stressors on muscle pain [9].

Poor working conditions bear some of the responsibilities of LBP in nursing personnel. Nurses in any department of a hospital are often administered with many unplanned works with varied tasks and responsibilities [10]. It is reported a strong association with LBP in nurses and postures in physical work such as the manual lifting of heavy objects or transferring patients and medical equipment [11, 12]. Many of the previous studies conducted to identify the roles of individual and occupational factors on LBP among nurses [13–16]. Serranheira et al. [17] described the prevalence increases and peaks between the ages of 35 and 55 years. Besides, lack of physical exercise is an important associated risk factor of LBP among the nurses. Lack of exercise renders inadequate flexibility and weak muscles in the back, pelvis, and thighs, causing increases of LBP [18–20]. Moreover, a number of studies indicated an association between work-related factors and LBP among the nursing staffs [14, 21, 22].

Nurses in developing countries have a higher incidence of occupation-related back pain due to lack of equipment and designs of work [4–6]. The intensity of LBP has rarely been taken into consideration in Bangladesh. It is important to find the occupational risk factors of LBP among nurses that will help to take necessary steps to minimize the risk of LBP and ensure safety occupational environment. We pay attention to different measures of LBP following Ozguler et al. [23]. To contribute to the present knowledge of LBP, we have carried out a cross-sectional study with the aim of identifying occupational risk factors in the Bangladeshi female working nurses.

## Data and methods

### The data

A cross-sectional study was conducted at Sylhet MAG Osmani Medical College & Hospital (public) and Jalalabad Ragib Rabeya Medical College & Hospital (private) in Sylhet, Bangladesh. On November 10, 2016, we found there was a total of 453 registered female nurses working in the two tertiary hospitals. Among which 245 and 208 female nurses were working in the public and private hospitals, respectively. As there are currently no studies that report the prevalence of LBP among nurses in Bangladesh, we consider the prevalence of LBP is 50% to estimate the sample size. At a confidence level of 95%, and margin error of 5%, the maximum sample size was calculated as 384 nurses. Using the finite population correction we have the required sample size is $\frac{384}{1 + 384/453}$ i.e. 233.

### Low back pain measures

Information on LBP was collected using a questionnaire with a diagram of the lower back area. We measured LBP by asking four questions following Feng et al. [16]. The first question about LBP (primarily pain, numbness, tingling, aching, stiffness, or burning of the lower back area) considers the pain that lasts for at least 1 day during the past 12 months. The other three questions of LBP measures are a chronic pain (daily pain for at least 3 months), intense pain (an intensity of pain score 6 and above on visual an analogue scale from 0 to 9) and seeking medical care (visit to a doctor or physiotherapist because of LBP during the last 12 months). Thus, a total of four different measures were used to define LBP.

### Independent variables

We designed a questionnaire to obtain information on date of birth, height, weight, date of survey, marital status (single or married), parity (yes or no), hospital (public or private), designation (head nurse or ordinary nurse), department (medicine, surgery, pediatric and obstetrics and gynecology), monthly family income (<25,000 BDT, 25,000–35,000 BDT and >35,000 BDT), average daily sleep duration, weekly working hours(≤40 or >40 h), prolong standing (1–2 or 2–3 h), manual lifting (yes or no), enough supporting staffs (yes or no) and regular physical exercise (yes or no). The duration of working was categorized as <5, 5–10 and >10 years. The overall job satisfaction was assessed by a question “Considering everything (coworkers, supervisor, availability of equipments) in your department, how satisfied are you with your job?”. The question was rated on a five-point Likert scale, ranging from 1 (strongly agree) to 5 (strongly disagree). We classify the sleep duration as short sleep duration (<6 h) and adequate sleep duration (≥6 h). We consider age in years from the date of births and we calculate body mass index (BMI) from height and weight. The age and BMI were managed as continuous variables. From December to January 2015, inspectors who were familiar with the questionnaire went to the two tertiary hospitals and taking face-to-face interview from the female nurses who are currently working in those hospitals. Any question or confusion from the participants was clarified to ensure that everyone understood all of the items. The complete questionnaires were reviewed for quality control.

### Statistical analysis

We analyzed the data using software R. We calculated the descriptive statistics for all of the variables which include continuous variables (presented as a mean and standard deviation) and categorical variables (presented as frequencies and percentages). The association between potential risk factors and LBP measures were modeled by separate multivariate logistic regressions with a backward selection procedure. The results were reported by adjusted odds ratios (ORs) and corresponded 95% confidence intervals (CIs). A *p* value less than 0.05 was considered statistically significant. Later, we investigate whether age is potential for confounding in the apparent relationship between an occupational factor and any of LBP measures. We found age relates to both LBP and any of the factors like designation, duration of working hours, BMI, parity, etc. Therefore to avoid biased results when examining the association between factors and LBP, we stratify age into two groups and considered modeling strategies with the nurses who are less than 40 years old.

## Results

### Characteristics of the study participants

The data comprised of 229 registered female working nurses from two tertiary hospitals: one of which is public and another one is private. The nurses were aged 22–56 years (mean 33.54 years). The baseline characteristics of the participants, such as designation, marital status, parity, family income, duration of working hours, prolong standing, manual lifting, supporting staff, physical exercise, sleeping hours and job satisfaction, are given in Table 1. The four different LBP measures are indicated a varied range of prevalence rates which is shown in Table 1. The most prevalence measure (72.9%) was pain that lasts for at least 1 day, which represents a broad category. Moreover, 31.8% nurses were found with chronic LBP; 36.2% reported having sought medical care in the past 12 months because of LBP, and 24.4% complained to suffer from intense pain. Among the nurses 73.8% were married, 59.8% had no children, 20.5% worked at least 10 years on the current job, and 59.3% worked overtime (i..e., >40 h per week). Compared with the private hospital, the tertiary public hospital had a higher prevalence of LBP. The age and BMI of the nurses are considered as continuous variables, and the mean and standard deviation are given in Table 2. The Table 2 shows that the average age of nurses who were suffering from chronic LBP is 37.7 (SD = 7.8) years. It appears from the Table 2 that older female nurses suffered more with LBP compared to younger female nurses (e.g., the average age of the nurses having chronic pain is 37.79 years whereas the average age is 31.55 years for the nurses who don’t have chronic pain). The average BMI of the nurses with chronic LBP was found 24.3 (SD = 3.0) kg/m^2^. The BMI indicates that weighted female nurses complained more on LBP compared to those who have less BMI (e.g., the average BMI is 24.36 kg/m^2^ for the nurses having chronic pain whereas the value is 22.99 kg/m^2^ for the group of no chronic pain).

**Table 1:** The baseline characteristics of the participants (n = 229)

Variables	Categories	LBP for ≥1 day (n = 167, 72.9%)	Chronic LBP (n = 73, 31.9%)	Intense pain (n = 56, 24.5%)	Seeking medical care (n = 83, 36.2%)
		Yes	Yes	Yes	Yes
Tertiary hospital	Private (105)	67 (63.8%)	14 (13.3%)	10 (9.5%)	30 (28.6%)
	Public (124)	100 (80.7%)	59 (47.6%)	46 (37.1%)	53 (43.7%)
Designation	Head nurse (55)	17 (30.9%)	9 (16.4%)	8 (14.6%)	12 (21.8%)
	Ordinary nurse (174)	150 (86.2%)	64 (36.8%)	52 (29.89%)	71 (40.8%)
Marital status	Single (60)	38 (63.3%)	8 (13.3%)	4 (6.66%)	15 (25.00%)
	Married (169)	129 (76.3%)	65 (38.6%)	52 (30.7%)	68 (40.3%)
Parity	No (92)	55 (59.8%)	17 (18.5%)	6 (6.5%)	24 (26.1%)
	Yes (137)	112 (81.8%)	56 (40.9%)	50 (36.5%)	59 (43.1%)
Family income	<25,000 TK (38)	28 (73.68%)	13(34.2%)	8 (21.1%)	12 (31.9%)
	25,000–35,000 TK (84)	58 (69.1%)	21 (25.0%)	12 (14.3%)	26 (30.9%)
	>35,000 TK (107)	81 (75.7%)	39 (36.5%)	22 (20.6%)	45 (42.1%)
Duration of working	<5 years (143)	102 (71.3%)	40 (27.9%)	31 (21.7%)	48 (33.6%)
	5–10 years (39)	24 (61.4%)	4 (10.3%)	3 (7.7%)	10 (25.6%)
	>10 years (47)	41 (87.3%)	29 (61.7%)	22 (46.8%)	25 (53.2%)
Weekly working hours	Overtime (93)	76 (81.7%)	37 (39.8%)	21 (22.6%)	42 (45.2%)
	Regular (136)	91 (66.9%)	36 (26.5%)	35 (25.7%)	41 (30.2%)
Prolong standing	1–2 h (128)	86 (67.2%)	29 (22.7%)	25 (19.5%)	38 (29.7%)
	2–3 h (101)	81 (80.2%)	44 (43.6%)	31 (30.7%)	45 (44.6%)
Manual lifting	Yes (176)	127 (72.2%)	60 (34.1%)	49 (27.8%)	67 (38.1%)
	No (53)	40 (7.5%)	13 (24.5%)	7 (13.2%)	16 (30.2%)
Supporting staff	Yes (94)	44 (47.3%)	15 (16.2%)	13 (14.0%)	21 (22.6%)
	No (135)	122 (90.4%)	58 (43.0%)	43 (31.9%)	62 (45.9%)
Physical exercise	Yes (36)	26 (72.2%)	10 (27.8%)	8 (22.2%)	9 (25.0%)
	No (193)	141 (73.1%)	58 (30.1%)	48 (24.8%)	74 (38.3%)
Sleeping hours	<6 h (118)	96 (81.4%)	53 (44.9%)	40 (33.9%)	55 (46.6%)
	≥6 h (111)	71 (64.0%)	20 (18.0%)	16 (14.4%)	28 (25.2%)
Job satisfaction	Strongly agree (47)	38 (80.9%)	21 (44.7%)	18 (38.3%)	22 (46.8%)
	Agree (123)	92 (74.8%)	44 (35.7%)	30 (24.4%)	45 (36.6 s %)
	Neither (59)	37 (62.7%)	8 (13.6%)	8 (13.6%)	16 (27.1%)

**Table 2:** Mean (standard deviation) of age and BMI corresponds to LBP measurements

	LBP lasted for ≥1 day		Chronic LBP		Intense pain		Seeking medical care	
	Yes	No	Yes	No	Yes	No	Yes	No
Age (years)	34.72 (8.2)	30.37 (6.6)	37.79 (7.8)	31.55 (7.3)	37.79 (7.4)	32.26 (7.8)	37.79 (7.7)	32.54 (8.04)
BMI (kg/m^2^)	23.95 (3.4)	22.00 (2.9)	24.36 (3.0)	22.99 (3.5)	24.98 (3.3)	22.92 (3.3)	23.86 (23.1)	3.05 (3.60)

### Significant risk factors for different LBP measures

We fit multivariable backward stepwise logistic regression models with each of LBP measures after adjusting all the occupational and individual risk factors. Table 3 shows the results of significant factors associated with each of the four measures of LBP. It appears that the different measures of LBP were found to be associated with different potential factors. Three occupational factors that include lack of supporting staffs (OR = 2.72), working overtime (OR = 1/0.38, i.e., 2.63) and having manual lifting in the daily job (OR = 1/0.365, i.e., 2.74) were found an association with different measures of LBP. The results indicate that the nurses in a department with the lack of supporting staffs are 2.74 times more likely to have the chronic back pain than the nurses who are working with adequate supporting staffs. Moreover, the nurses working overtime are 2.63 times more likely to have the chronic pain than the nurses working a regular hours. Age (OR = 1.08) is also found significant to develop chronic pain which indicates 8% increase in the risk of having chronic back pain by 1 year increase of age. Another individual factor parity is found positively associated with intense pain and the odds ratio indicates that nurses with children are 4.07 times more likely to have intense back pain in their lifetime than the nurses who do not have children. We considered age as a confounding variable because it influences both LBP and the other independent variables like designation, duration of working hours, BMI, parity, etc. To avoid age as a confounding variable, we considered data from nurses less than 40 years of age. We fit a multivariate backward stepwise logistic regression model with each of the four LBP measures after adjusting all the occupational and individual risk factors. Table 4 shows the results of significant factors associated with each of the four measures of LBP with nurses less than 40 years of age. The results indicate that lack of supporting staffs is positively related with each of the LBP measures. Two other work-related factors that include manual lifting of patients or equipments in their work environment and working overtime are found associated with LBP. Public hospital (OR = 4.88) nurses were found more affected by the LBP, especially with chronic pain. The results from the Table 4 are found consistent with the results of Table 3. Therefore, it appears that mainly three occupational factors, lack of supporting staffs, manual lifting and overtime working hours, are found significant to develop LBP.

**Table 3:** Association of different LBP related measures with risk factors identified from multivariate backward stepwise logistic regression (Overall)

LBP measures	Significant risk factors	Reference	OR	LCL	UCL	*p* value
LBP for ≥1 day	Age		1.094	1.003	1.202	0.048
	Hospital-public	Private	0.268	0.062	1.098	0.071
	parity-yes	No	3.342	1.148	9.947	0.027
	Working hours-regular	Overtime	0.475	0.224	0.974	0.046
	Supporting staff-no	Yes	10.67	4.884	25.114	<0.001
Chronic LBP	Age		1.08	1.01	1.159	0.026
	Working hours-regular	Overtime	0.38	0.181	0.774	0.008
	Manual lifting-no	Yes	0.365	0.149	0.842	0.022
	Supporting staff-no	Yes	2.722	1.301	5.914	0.009
Intense pain	Parity-yes	No	4.068	1.118	20.238	0.05
	Manual lifting-no	Yes	0.29	0.102	0.731	0.012
Seeking medical care	Working hours-regular	Overtime	0.485	0.257	0.904	0.023
	Supporting staff-no	Yes	2.788	1.431	5.596	0.003
	Manual lifting-no	Yes	0.487	0.225	1.008	0.058

**Table 4:** Association of different LBP related measures with risk factors identified from multivariate backward stepwise logistic regression when age ≤ 40 years

LBP measures	Significant risk factors	Reference	OR	LCL	UCL	*p* value
LBP for ≥1 day	Parity-yes	No	3.088	1.002	9.91	0.052
	Working hours-regular	Overtime	0.426	0.182	0.955	0.042
	Supporting staff-no	Yes	7.158	3.186	17.234	<0.001
Chronic LBP	Hospital-public	Private	4.889	1.265	20.759	0.024
	Working hours-regular	Overtime	0.265	0.108	0.622	0.002
	Manual lifting-no	Yes	0.366	0.119	0.988	0.052
	Supporting staff-no	Yes	2.371	0.986	5.961	0.057
Intense pain	Manual lifting-no	Yes	0.189	3.951	0.655	0.017
Seeked medical care	Working hours-regular	Overtime	0.399	0.189	0.828	0.014
	Supporting staff-no	Yes	2.26	1.05	4.998	0.039

## Discussions

Nurses in hospitals of Bangladesh are commonly found with low back pain that occurs not because of accidents, but because of few occupational factors. We study the LBP among nursing staffs because of its professional, economic and social burden. Without knowing the occupational factors that cause LBP might severely hamper effective prevention and management. Based on the female nurses in the previous 12 months, the prevalence rates for pain lasts for at least 1 day, intense pain, chronic pain, and seeking medical care were 73.0, 24.4, 31.8, and 36.2%, respectively. The results indicate higher prevalence of LBP in Bangladeshi nurses than the Taiwanese female nurses where the prevalence rates for pain lasting for at least one day, seeking of medical care, intense pain, sick leave, and chronic pain were 66.0, 43.9, 38.1, 10.7, and 8.6%, respectively [16]. This result indicates that the prevalence rate of LBP among nurses in Bangladesh is high and more active steps need to be taken to address this problem.

In this study, we found three occupational factors are mainly associated with LBP measures. The significant risk factors are a lack of supporting staff, manual lifting and overtime working hours, whereas some demographic factors like age and parity were related with different LBP measures. These findings are found consistent with previous findings [24, 25]. Therefore, it is important to increase nursing staffs by taking consideration of nurse to patient ratio. Insufficient supporting staffs in a hospital might increase the frequency of manual handling per nurse as well work overtime and in turn lead to greater risk of LBP. One of the limitations of this study to use of cross-sectional design which might create exaggeration of some relationships noted in this study since the study subjects with LBP might be inclined to over-report their psychosocial load or physical exertion. The study involves only two tertiary hospitals, which means additional studies are needed to draw a more precise conclusion in developing LBP among the nurses of Bangladesh.

## Conclusion

The study indicates that the prevalence rate of LBP among the female nurses in Bangladesh is high and more operative steps should be taken to address this problem. The attention should focus on increase supporting staffs, reduce weekly working hours and lessen the frequency of manual lifting in the work environment.

## Abbreviations

LBP: low back pain
BMI: body mass index
OR: odds ratio
CI: confidence interval
SD: standard deviation
